# Beyond division and morphogenesis: Considering the emerging roles of septins in plasma membrane homeostasis and cell wall integrity in human fungal pathogens

**DOI:** 10.1371/journal.ppat.1013226

**Published:** 2025-06-17

**Authors:** Stephani Martinez Barrera, Lukasz Kozubowski

**Affiliations:** 1 Department of Genetics and Biochemistry, Eukaryotic Pathogens Innovation Center, Clemson University, Clemson, South Carolina, United States of America; 2 Department of Neuroscience, Washington University School of Medicine, Saint Louis, Missouri, United States of America; University of Maryland, Baltimore, UNITED STATES OF AMERICA

Septins comprise a highly conserved family of guanine nucleotide-binding, filament-forming proteins found in animals, fungi, protists, and algae, but absent in land plants [1–3]. Septins were discovered by electron microscopy as a set of 10-nanometer filaments surrounding the junction between the mother cell and the bud in *Saccharomyces cerevisiae* and were named for their role in cytokinesis and formation of the septum [4–17]. The two main features of septins are the assembly into higher-order filamentous structures which facilitates scaffolding other proteins and the ability to interact with membranes which allows the septins to act as membrane diffusion barriers. Investigating those features in various organisms linked the septins to multitude of cellular processes beyond cytokinesis, including morphogenesis, cell polarity, cell cycle regulation, and aging [5,14,18–27].

The significance of GTP binding and hydrolysis in septin function is multifaceted, impacting both septin structure and dynamics as well as their interaction with other cellular components [25,28–33]. Unlike classical regulatory GTPases such as Ras or Rho, which act as molecular switches to regulate downstream signaling pathways, septins employ the bound nucleotide mainly to stabilize or remodel the end-to-end interfaces within hetero-oligomeric rods that assemble into rings and ordered gauzes [25,34,35].

Given their widespread impact on cellular physiology, it is not surprising that septins are important for fungal pathogenesis (Fig 1 and Table 1). Septin mutants show reduced fungal virulence in both plants and animals [13,36–39] except for *Aspergillus fumigatus* [23,40]. While individual septins may not be necessary for virulence of *A. fumigatus*, the entire septin complex may still be required for full virulence of this pathogen [40]. Very interestingly, pathogenesis connection relates to both the pathogen and the host. For instance, mammalian septins facilitate the invasion of the host cells by *Listeria monocytogenes* [41], while septins in *Magnaporthe oryzae* are essential for the fungal invasion and subsequent infection of the plant [36]. What unifies the roles of septins in pathogenesis appears to be their association with the biological membranes [42].

**Table 1:** Functions of septins relevant to human fungal infections.

Organism	Mutants	Virulence	Function	References
***C. albicans***	*cdc3*Δ *cdc10*Δ *cdc11*Δ *cdc12*Δ	*cdc10*Δ & *cdc11*Δ result in reduced invasive growth. In addition, *cdc10*Δ & *cdc11*Δ strains exhibit attenuated virulence in animal models of infection.	Hyphal growth (site of bud emergence and at the base of hyphae), Protein scaffolds at cell division site, cell wall stress response	[69–71,84,85]
***A. fumigatus***	*aspA*^*cdc11*^Δ *aspB*^*cdc3*^Δ *aspC*^*cdc12*^Δ *aspD*^*cdc10*^Δ *aspE*Δ	*aspA*^*cdc11*^Δ, *aspB*^*cdc3*^Δ & *aspC*^*cdc12*^Δ are hypervirulent in *G. mellonella*	Septation, conidiation, cell wall stress response, ascopore formation, morphogenesis	[40,86]
***A. nidulans*** ^*^	*aspA*^*cdc11*^Δ *aspB*^*cdc3*^Δ *aspC*^*cdc12*^Δ *aspD*^*cdc10*^Δ *aspE*Δ	Not investigated	Nuclear division, septation, conidiation, negative regulation of new grow foci, coordination of cell wall integrity, and lipid metabolism in a sphingolipid-dependent process	[48,87–89]
***C. neoformans***	*cdc3*Δ *cdc10*Δ *cdc11*Δ *cdc12*Δ	*cdc3*Δ & *cdc12*Δ result in reduced virulence in a *G. mellonella*	Cell division site, sexual reproduction (morphogenesis), nuclei distribution, cell wall stress response, pathogenicity	[39,90]

* *Aspergillus nidulans* is a filamentous fungal model and not a traditionally classified human fungal pathogen. However, it is included in this table because recent studies have shown that *A. nidulans* can cause invasive aspergillosis (IA), particularly in patients with chronic granulomatous disease (CGD), highlighting its emerging clinical relevance [91,92].

**Fig 1:**
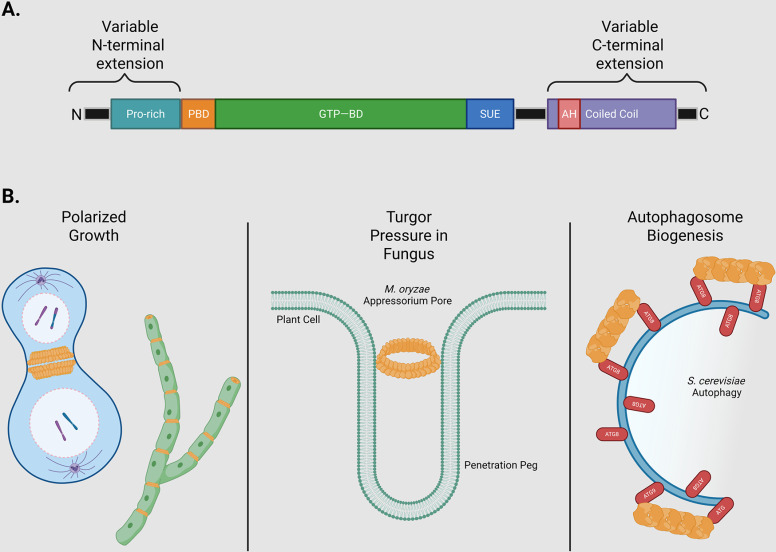
Features and functions of fungal septins related to pathogenesis. **(A)** Schematic showing the structural domains of septin proteins. All septins contain a GTP-binding domain (GTP-BD), the septin unique element (SUE), and a phosphoinositide-binding polybasic domain (PBD) [25,93]. The GTP binding and hydrolysis plays an important role in the assembly of the septin higher order structures. All septins contain N- and C-terminal extensions (NTE, CTE) of variable length [93,94]. The CTE contains one or more coiled-coil regions, which vary across septins [25]. Septin Cdc10 is the only one that lacks this feature while Cdc12 is the only septin that contains an amphipathic helix (AH) domain [13,22,56]. Membrane binding is mediated by AH and PBD motifs [25,56]. The N-terminus consists of a proline-rich domain, for interaction with other septins [25]. **(B)** Functions of septins in fungal pathogens. Septins interact with lipids or proteins at membranes and are enriched at positively curved membranes by binding to phosphoinositides, where they contribute to polarized growth. In yeast cells, the membrane recruitment of septins follows high concentrations of Cdc42 that cycles from inactive to active states, directing further outgrowth of the bud [13]. In addition to polarized growth, septins contribute in cytokinesis, in both yeasts and hyphae, by coordinating cell division. Furthermore, septins can act as barriers or scaffolds at membranes [13,75]. In *Saccharomyces cerevisiae*, septin proteins accumulate at the cortex of the mother–bud neck. Early in bud formation, they assemble into filament arrays that run along the cell’s polarity axis. This filament framework arranges the formin Bnr1 and the F-BAR protein Hof1 into regularly spaced, pillar-like structures, which in turn organize actin cables that are evenly separated and directed toward the developing bud [47,75]. Once the cell enters mitosis, the septin scaffold recruits myosin II, initiating assembly of the actomyosin ring (AMR). At the start of cytokinesis, septins link to the AMR indirectly through Hof1, which binds both septins and myosin II. At the onset of cytokinesis, the septin filaments undergo ~90° reorientation and the septin collar separates into two rings that flank the AMR [75,94]. In the plant pathogen *Magnaporthe oryzae*, septins regulate pathogen invasion [18,24,94]. Positioned at the base of the appressorium, a pressurized hyphal structure used to breach the host cuticle, a septin collar directs the construction of a ring-shaped (toroidal) actin network. It does so by confining the I-BAR protein Rvs167 and the WASP-related factor Las17 within its perimeter, while also acting as a scaffold for the ERM protein Tea1, which strengthens the connection between actin filaments and the membrane [24,36,43]. Septins are also regulators of autophagy. In *S. cerevisiae*, septins are involved in forming ring-like structures at the pre-autophagosomal structure (PAS), which is where autophagosomes begin to form [82,83]. These structures can interact with autophagy proteins like Atg8 and Atg9, which are crucial for autophagosome biogenesis. Created in BioRender. Martinez Barrera, S. (2025) https://BioRender.com/2pcxcq0.

What specific functions of septins contribute to pathogenesis? Septins participate in cytokinesis which is essential for proliferation. *Cryptococcus neoformans* lacking septin Cdc3 or Cdc12 proliferates under no-stress conditions (exhibiting partly compromised septa), potentially due to compensatory mechanisms [39]. However, *C. neoformans* septin mutants fail to proliferate at host temperature, suggesting that at 37 °C, the putative compensatory mechanisms are not sufficient to sustain cytokinesis [39]. This may be a common contribution of septins to virulence in all fungal pathogens. Similarly, established functions of septins in polarity and cell cycle regulation may explain their important roles in fungal virulence. Those roles are consistent with associations of septins with cytoskeletal machinery including actin and microtubules, which may be critical for morphological transitions necessary for virulence [13,18,22,28,36,43–47]. In addition, the roles of septins in polar growth and the functions in cell wall (CW) synthesis and integrity, which is the focus of this review, are likely interlinked.

Considering the functional connection between the plasma membrane (PM) and the CW, the PM association of septins is predicted to influence CW composition, which collectively should have an impact on fungal pathogenesis, especially considering stress encountered in the host. However, the mechanisms related to PM homeostasis and cell wall integrity (CWI) that may be responsible for septin contribution to the virulence of many fungal pathogens remain largely unexplored [48].

## Q1: What is known about interactions of septins with biological membranes?

Septins associate with membrane phospholipids with preference for phosphatidylinositol (4,5) bisphosphate (PI(4,5)P_2_) [49]. Typically, higher-order septin assemblies localize at sites of membrane curvature, such as the mother-bud neck in yeast or at the base of hyphal branches in filamentous fungi [28]. It is worth noting that septins also assemble at the shmoo base in *S. cerevisiae* exposed to mating pheromone [50]. These studies provided one of the earliest demonstrations that septins can function outside their classic role in cytokinesis. Because pheromone treatment halts cells in G1, the canonical septin ring does not assemble; instead, an alternative septin structure at the shmoo base is formed [51,52].

Septins association with membranes occurs as a stepwise process as septins initially diffuse, collide with one another, and anneal into higher-order assemblies [19,28,53,54]. Septins intrinsically ‘sense’ positive membrane curvature at the micron scale [42] by a mechanism that remains a subject of inquiry [28,55]. Certain septin subunits contain an amphipathic helix (AH) domain at their C-terminus, which is both necessary and sufficient to discern membrane curvature, as is found in many nanometer-scale curvature sensors [3,56,57] (Fig 1A). The AH domain is characterized by polar residues on one face and hydrophobic residues on the other, enabling it to interact with the membrane. It is theorized that membrane curvature induces lipid-packing defects, thereby creating binding sites for AH domains [28,55,58].

Septins reshape membranes in giant unilamellar vesicles *in vitro* [59]. Remarkably, in this study septins “avoided” positive curvature and preferentially assembled at the crest of supported lipid monolayer ridges or in the troughs with negative curvature [59]. Several experimental factors may account for the observed differences in septin filament alignment, including the use of lipid bilayers versus monolayers, lipid composition, and the topology of the membrane support substrate [28,56,59]. In another study, septins prevented temperature-induced changes in the composition of the lipid bilayer *in vitro* [60]. Thus, the physical interaction of septins with the membranes involves reciprocal impact with the membrane curvature influencing the degree of association and the septins having an impact on membrane biophysical properties.

## Q2: What roles do septins play in fungal cell wall integrity?

In fungi, the CWI pathway plays a crucial role in withstanding external stress, which is critical for survival in hostile environments [61,62]. The CW facilitates interaction with the external environment since some of its components are receptors and adhesins that can drive the host’s immune response or promote fungal growth, dissemination, and virulence [61–65].

The connection between CW and septin function is well-established. In *S. cerevisiae*, septin complex plays a crucial role in the deposition of CW chitin at the incipient bud site [13,66–68]. The synthesis of this chitin ring depends on the activity of chitin synthase III (Chs3) and its proper localization, which is tethered to the septins by a hierarchy of proteins [13,68].

Studies in *Candida albicans* and *Aspergillus nidulans* have linked septins to the CWI pathway and the mitogen-activated protein kinase (MAPK) signaling pathway [48,69–71]. In *C. albicans*, the plant defensin RsAFP2 interacts with fungal glucosylceramides, leading to ceramide accumulation, mislocalization of septins, and abnormal CW morphology [69]. The study identifies several RsAFP2-tolerance genes involved in CWI and septin ring formation [69].

Exposure of *C. albicans* to caspofungin, which inhibits the β-1,3-D-glucan synthesis and creates CW stress, leads to the accumulation of septins at PM sites common with PI(4,5)P_2_ [70,71]. Redistribution of septins is highly dynamic and corelates with the activation of the PKC-Mapk1 pathway [71]. In addition, the eisosome protein Sur7 restrains plasma-membrane PI(4,5)P_2_ by modulating the Inp family of 5′ phosphatases. When the CW is damaged, membrane stress disables Sur7’s control, allowing PI(4,5)P_2_ to accumulate in discrete patches [72]. These PI(4,5)P_2_ patches recruit septins, which stimulate localized cell-wall synthesis to seal the lesion, after which eisosome organization and PI(4,5)P_2_ levels return to their baseline state [73]. Mutants lacking Sur7, core eisosome proteins (Pil1/Lsp1), or the Inp phosphatases, cannot down-regulate PI(4,5)P_2_, so they chronically behave as though the CW were damaged, accumulating excess inward cell-wall material despite the absence of actual injury [72,73].

*A. fumigatus* septin deletion strains are hypersensitive to CW inhibitors, indicating potential CW defects [40]. *A. nidulans* core septin mutants also showed hypersensitivity to various CW-disturbing agents, while the noncore septin mutant showed only mild sensitivity [48]. Furthermore, *A. nidulans* septin mutants exhibited increased chitin content in comparison to wild-type, suggesting a compensatory mechanism during CW stress. A detailed analysis involving single and double septin mutants in *A. nidulans* suggests that core septins modulate the CWI pathway under CW stress [48].

Thus, septins play a role in maintaining CWI, but the precise mechanisms remain unclear. Septins may exert indirect effects through their interactions with other CW-associated proteins. For instance, their role in organizing the actin cytoskeleton could indirectly affect CW [45,74–77]. Understanding whether septins function as structural stabilizers or active participants in stress-responsive CW remodeling is key to unraveling their contribution to CWI.

## Q3: Are septins involved in the crosstalk between cell wall integrity and plasma membrane homeostasis?

PI(4,5)P_2_ plays a crucial role in maintaining PM stability in *C. albicans* through its involvement in activating the Pkc-Mapk CWI pathway, which is necessary for the synthesis and deposition of chitin [78]. Mutations in genes such as Irs4 and Inp51, which are involved in the regulation of PI(4,5)P_2_ levels, lead to elevated PI(4,5)P_2_ and result in the formation of discrete patches on the PM [71,79]. These patches are associated with CW derangements, and PM invaginations. The aberrant distribution of PI(4,5)P_2_ in these mutants suggests a disruption in the normal PM structure and function [70,71]. Septins colocalize with PI(4,5)P_2_ and chitin in these patches, suggesting a functional interaction between PI(4,5)P_2_ and septins in maintaining CWI under stress conditions [70,71], a possibility consistent with the scaffolding function of septins [80]. It has been hypothesized that the role of PI(4,5)P_2_ in the CWI of *C. albicans* is potentially mediated by septin proteins and/or their respective regulators [71].

In *A. nidulans*, septins monitor lipid microdomain composition and/or organization, which is crucial for maintaining PM stability and signaling through the Mapk pathway [48]. Core septins mislocalized after treatment with sphingolipid-disrupting agents, but not after ergosterol-disrupting agents, suggesting a specific role in sphingolipid metabolism and its interaction with the Mapk pathway [48]. Remarkably, septin-null mutants did not exhibit significant sensitivity to inhibitors of Ca2+/calcineurin, cAMP-PKA, and TOR pathways, which suggests a more specific interaction of septins with the CWI pathway [48]. Thus, in *A. nidulans*, septins scaffold CWI machinery, monitor lipid microdomain composition, and signal sphingolipid status to the CWI pathway, which is essential for maintaining CWI and responding to CW stress [48].

## Q4: What does the future hold for research on septins contributions to stress response in fungal pathogens?

Septins play essential roles in fungal biology, yet their connection to stress response remains a largely underexplored frontier. Their involvement in fungal PM and CWI suggests that they serve as key players in adapting to environmental challenges. Septins are known to associate with membranes and preferentially localize to regions of membrane curvature, playing a role in morphogenesis. However, their potential influence on the physical properties of membranes *in vivo* remains largely underexplored. One intriguing possibility is that septins help maintain PM stability during stress conditions, acting as a protective scaffold to prevent mechanical failure. The effects of septins on membrane-associated structures suggest functions crucial to stress response that are beyond acting as diffusion barriers. Are their roles in CW and membrane stability independent, or are these effects a consequence of a crosstalk? Do septins participate in sensing environment by acting as effectors in stress adaptation, and/or do they provide structural support? Given their presence at key cellular interfaces, septins may act as signaling hubs for stress response pathways. Recent reports link septins to autophagy which may be one of the key functions relevant to host invasion [81–83]. Furthermore, septins functions in cytokinesis may be more closely tied to stress adaptation than previously thought, particularly in ensuring robust PM and CWI at the division site when the cell encounters stress. Addressing these questions will not only enhance our understanding of the biology of septins but may also reveal their potential as targets for antifungal strategies.
